# Hair as a Biomarker of Long Term Mercury Exposure in Brazilian Amazon: A Systematic Review

**DOI:** 10.3390/ijerph15030500

**Published:** 2018-03-12

**Authors:** Nathália Santos Serrão de Castro, Marcelo de Oliveira Lima

**Affiliations:** 1 Centre of Research and Extension, Metropolitan College of Amazon (FAMAZ), Visconde de Souza Franco Avenue, 72, Belém-Pará 66053-000, Brazil; 2 Environmental Section, Evandro Chagas Institute, BR-316, s/n, Ananindeua-Pará 67030-000, Brazil; marcelolima@iec.pa.gov.br

**Keywords:** mercury, methylmercury, Amazon, hair Amazonia

## Abstract

Many studies have assessed mercury (Hg) exposure in the Amazonian population. This article performs a literature search of the studies that used hair as a biomarker of Hg exposure in the Brazilian Amazonian population. The search covered the period from 1996 to 2016 and included articles which matched the following criteria: (1) articles related to Hg exposure into Brazilian Amazon; (2) articles that used hair as a biomarker of Hg exposure; (3) articles that used analytical tools to measure the Hg content on hair and (4) articles that presented arithmetic mean and/or minimum and maximum values of Hg. 36 studies were selected. The findings show that most of the studies were performed along margins of important rivers, such as Negro, Tapajós and Madeira. All the population presented mean levels of Hg on hair above 6 µg g^−1^ and general population, adults, not determined and men presented levels of Hg on hair above 10 µg g^−1^. The results show that most of the studies were performed by Brazilian institutions/researchers and the majority was performed in the State of Pará. The present study identified that Amazonian population has long-term been exposed to Hg. In terms of future perspectives, this study suggests the implementation of a strategic plan for environmental health surveillance in the region in order to promote health and benefit Amazonian population.

## 1. Background

The health effects of methylmercury (MeHg) exposure have been investigated since the accident that occurred in Minamata Bay, Japan. The clinical features of MeHg poisoning were classified as acute or chronic, based on the symptoms observed in patients living around the bay in the vicinity of the pollution source (a factory) and in patients living on the coast of the Shiranui Sea; both populations had consumed contaminated fish for almost 20 years [1].

Clinical studies of Japanese patients affected by dietary MeHg poisoning showed that mercury (Hg) had long-term effects on health. The patients from Goshoura Island, an area close to Minamata Bay, who had mean levels of total hair Hg of 37 µg g^−1^ in 1960 (*n* = 16) and 2.4 µg g^−1^ in 2002 (*n* = 23) showed persistent sensory disorders caused by their past history of MeHg exposure [2,3]. Evaluation of data from a population-based study performed in 1971 at Goshoura showed an increased incidence of neurological signs, such as ataxia (12%), dysarthria (5.9%), and paresthesia of extremities (5.7%) [4]. A study performed at Niigata, a town located along the Agano River that was affected by MeHg poisoning in 1965, showed that even people with chronic exposure to levels of Hg of less than 20 µg g^−1^ (*n* = 24) presented neurologic signs associated with MeHg poisoning [5]. Recently, a population-based study in Minamata and neighboring areas identified the association between neurological sign and the development of psychiatric symptoms.

In spite of the findings observed after the Minamata disaster, there is no clear consensus yet about a dose-response relationship between Hg exposure and health effects given that the genetic characteristics of the populations vary, the modes and times of exposure are diverse, and that life-styles and behavior can influence on the toxic effects of Hg exposure [6,7]. However, vulnerable populations (i.e., pregnant women, human fetuses, neonates, and children) are under the potential risks of Hg effects [8,9,10,11].

Analysis of scalp hair has been a valuable method used to assess the Hg exposure of different populations because hair is easy to collect, store, and manipulate [12]. Hair Hg levels strongly correlate with an individual’s dietary intake of MeHg. Moreover, its chemical stability facilitates retrospective studies [13]. However, Hg can be incorporated into hair in other ways, such as the sorption of volatile species (i.e., elemental Hg), and its level can be affected by hair color and growth rates, which are considered to be pre-analytical sources of variation that may cause bias and misleading interpretations [14,15]. In spite of these limitations, the versatility provided by scalp hair for assessing Hg exposure, especially in remote areas, has been valuable to access the Hg exposure in different populations.

In Brazil, many studies had been performed in the Amazonian region and hair has been selected as a biomarker of Hg exposure. The present article performs a systematic review of publications that analyzed Hg on hair of different populations into Brazilian Amazon. The objective of this article is to provide an overview of long-term exposure to Hg into Brazilian Amazon, identify populations under risk of Hg effects and give future perspectives for environmental health surveillance for the region.

## 2. Methods

This systematic review was registered in PROSPERO (registration number CRD42017056584). The search was performed using the following electronic databases: Pubmed, EBSCO, VHL (Virtual Health Library) and Scielo. Both authors performed independently the virtual search for articles titles and abstract using the search strategy showed in the Table 1. The search covered the studies that were published in the period from 1996 to 2016. The present study followed the following inclusion criteria for articles: (1) articles related to Hg exposure into Brazilian Amazon; (2) articles that used hair as a biomarker of Hg exposure; (3) articles that used analytical tools to measure the Hg content on hair and (4) articles that presented arithmetic mean and/or minimum and maximum values of Hg. As exclusion criteria, the present study followed: (1) review articles of Hg exposure and (2) articles which methodology was not clear, such as sample size, locality and analytical tools. The potential studies were screened and duplicates were removed. Articles not excluded were read by the two authors who extracted in detail the geographic location where the study was performed, sample size and type, the age range and/or mean age of the populations, the first author of the study, the year of the publication, and the arithmetic mean of Hg observed.

The present study classified the type of the studied populations as follows: general population (0 to >60 years old), adult population (>15 years old), children (<15 years old), women, men, indigenous and not determined (nd) when the study did not identify the type and/or the age range of the target population.

According to the type of each population, the data of the mean level of Hg was used in order to determine a weighted average in which the number of individuals studied contributed equally to a final average (Equation (1)).

(1) WA=((X1×I1)+(X2×I2)+(Xn×In))/(ΣI1+I2+In)

where: *WA* = weighted average; *X* = arithmetic mean of Hg (µg g^−1^); *I* = Number of Individuals.

A cartographic quali-quantitative analysis of the data was performed. The classification of the populations determined for this study were considered for a qualitative evaluation and a quantitative analysis based on the degree of exposure followed the following criteria: lower (less than 2 µg g^−1^), medium (between 2 and 6 µg g^−1^) and higher (above 6 µg g^−1^).

The ArcMap 10.1 software (ESRI, Redlands, CA, USA) was used for georeferencing for studied where the geographic coordinates were not available. The geographic location was matched with the Brazilian Institute of Geography and Statistics (IBGE). The quantitative analysis was evaluated by a “choropleth map” where the differences of Hg exposure according to different populations and regions were shaded: yellow representing populations with low exposure to Hg and red high exposure.

Among the major source of risk of bias of the present study we can cite here:

(1) The period of the study.

The present study searched for studies that were published from 1996 to 2016 (20 years). Thus, the concentration of Hg on hair among the populations can vary, and, thus, the general visualization provided by the cartographic quali-quantitative analysis could not represent the current status.

(2) The methodologies for Hg measurement were not an exclusion or inclusion criteria.

Once the present study considered valid all the methodologies that measured Hg on hair, the results can not reflect a “gold standard” for Hg exposure in Amazon. Instead, all the selected articles were peer reviewed, thus the data could be considered to give an overview of long term Hg exposure in Amazonian population.

## 3. Results

**Figure 1:**
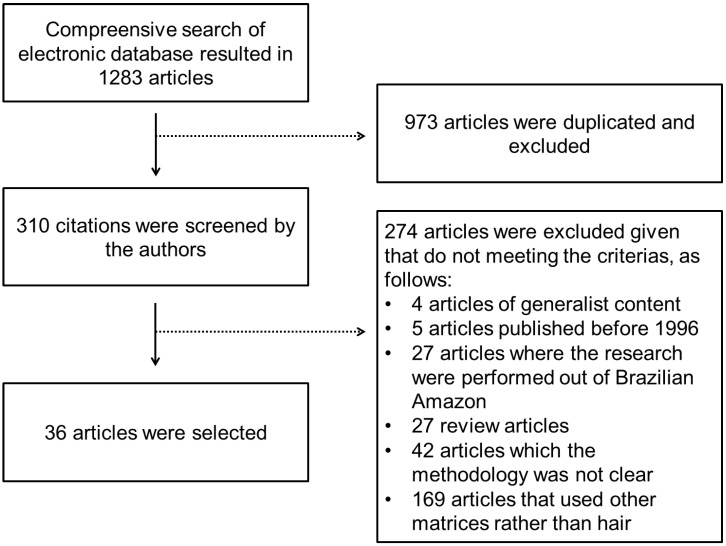
Study selection flowchart.

The database search identified 1283 articles, of which 973 were duplicated and, consequently, removed. The potential 310 articles were screened and 274 of them were removed given that did not attended to some inclusion/exclusion criteria: 169 used other matrices rather than hair, 42 which methodology was not clear, 27 were review articles, 27 were performed in other regions rather than Brazilian Amazon (7 in Peru, 6 in Bolivia, 4 in Ecuador, 3 in French Guiana, 1 in Venezuela, 1 in Suriname, 1 in Peru and Ecuador, 1 Faroe Island, 1 in Colombia, 1 in Africa and 1 in the Northwest of Brazil), 5 were published out of the period of investigation and 4 contained generalist subject about Hg exposure. The study selection is summarized in the flowchart described in Figure 1.

In this context, the present study comprised 36 articles, comprising a total of 11,827 individuals (Table 2). According to the year of publication, the articles were published as follows: three in 1998 [16,17,18], two in 1999 [19,20], three in 2000 [21,22,23], two in 2001 [24,25], one in 2002 [26], two in 2003 [27,28], four in 2005 [29,30,31,32], three in 2006 [33,34,35], two in 2007 [36,37], one in 2008 [38], one in 2009 [39], two in 2010 [40,41], three in 2012 [42,43,44], one in 2013 [45], one in 2014 [46], three in 2015 [47,48,49] and two in 2016 [50,51]. Most of the studies (61%) were performed exclusively by Brazilians Institution [16,18,20,21,22,24,28,29,30,31,32,33,34,37,41,42,43,44,45,46,49,51] and others (36%) had been performed in collaboration with them [17,19,23,25,26,35,36,38,39,40,47,48,50]. Only one study (3%) was developed for a foreign country solely [27].

There was a predominance of studies that were performed in the State of Pará (47%) [22,23,25,26,27,29,35,36,37,38,39,40,42,43,45,47,48]. Some studies had been performed in the State of Amazonas (22%) [17,19,24,30,33,41,44,49], Rondônia (14%) [18,28,46,50,51], Mato Grosso (8%) [21,31,32] and Amapá (3%) [20]. Two studies were performed using samples from populations from two different States: Pará and Rondônia (3%) [16] and Rondônia and Amazonas (3%) [34].

**Figure 2:**
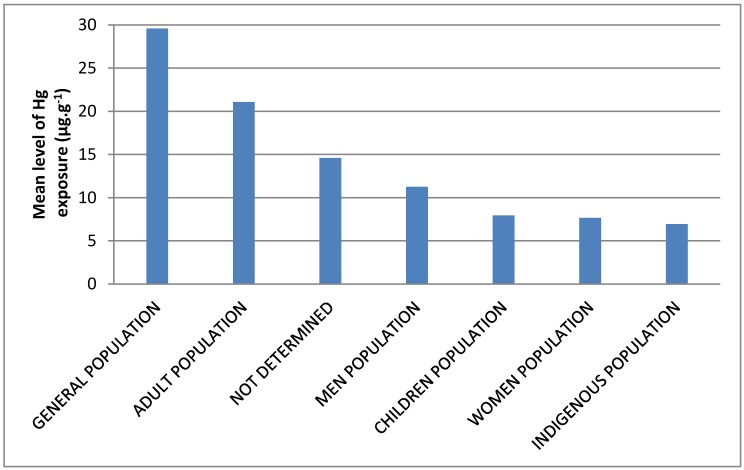
Mean level (µg g^−1^) of Hg exposure in populations of Brazilian Amazon according to the present study.

The results shows that general population presented the highest mean level of Hg exposure (29.59 µg g^−1^, ranging from 0.73 to 97.44 µg g^−1^), followed by adult population (21.08 µg g^−1^, ranging from 1.00 to 43.70 µg g^−1^), not determined (14.60 µg g^−1^, ranging from 1.00 to 62.76 µg g^−1^), men population (11.25 µg g^−1^, ranging from 11.00 to 11.50 µg g^−1^), children population (7.95 µg g^−1^, ranging from 1.11 to 22.00 µg g^−1^), women population (7.66 µg g^−1^, ranging from 0.60 to 14.30 µg g^−1^) and Indigenous population (6.95 µg g^−1^, ranging from 4.90 to 8.37 µg g^−1^) (Figure 2).

**Figure 3:**
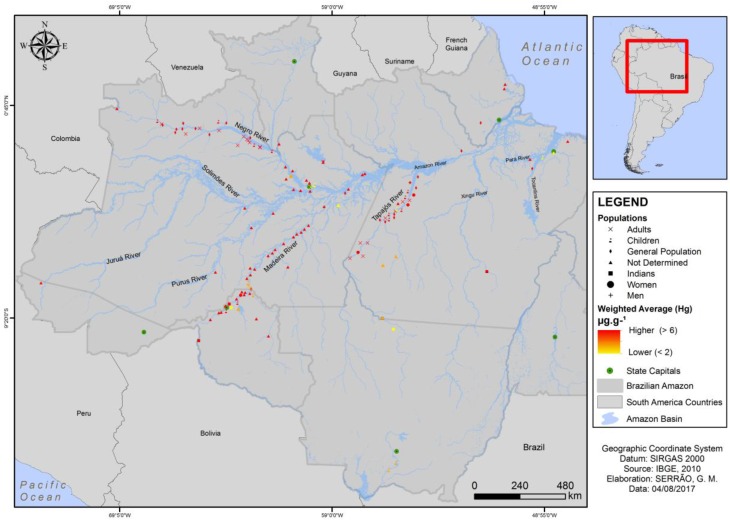
The georeferencing results showing a geospatial distribution of the Brazilian Amazonian populations and their respective degree of Hg exposure on hair.

The analysis of the georeferencing data is presented as a map which shows a geospatial distribution of the populations and their respective degree of Hg exposure. The results show that most of the studies were performed along the margins of the Amazonian rivers and that most of these populations are highly exposed (Figure 3).

## 4. Discussion

The theme of Hg exposure in Amazonian population is an intriguing form of environmental contamination for some reasons: (1) the mean level of Hg exposure in Amazonian population usually exceed the normal limit preconized by WHO [24,30,33,52,53,54]; (2) there is a dichotomy in the clinical findings in different Amazonian populations, where some studies associate Hg exposure with the development of clinical symptoms [35,55,56,57] while others do not [45,58,59,60,61] and (3) even “non-exposed” populations are at risk of Hg effects [58].

The present study performed a systematic review of Hg exposure in Brazilian Amazon population. The results show that the majority of the studies were performed by Brazilian Institutions and researchers, reflecting the low international insertion despite that Hg is a global problem [62]. The high number of studies that were performed in the State of Pará is in agreement with the high prevalence of gold miners in the region (legal and illegal miners), especially in the Tapajós Basin [26,63].

The analysis shows that most of the studies were performed along the margins of important rivers, such as Negro, Tapajós and Madeira Rivers. The results show that all the populations presented mean levels above 6 µg g^−1^ of Hg on hair and that general population, adults, not determined and men presented mean levels above 10 µg g^−1^ of Hg on hair. Thus, the findings support the idea that Amazonian population present (along the period of time covered by this study) mean levels of Hg above the normal limit preconized by WHO (1–2 µg g^−1^ and levels above 10 µg g^−1^ for daily fish consumers) [64]. Although, affirm that this population is under risk of Hg effect is premature. Discussions about this issue should be evaluated based on other studies.

As future perspectives, the present study suggests an implementation of a strategic plan for the region in order to promote health and benefit the population. As strategies, we propose:

(1) Increase the technical capacity for Hg determination in the region;
(2) Implement an environmental health surveillance program that considerers the Amazonian life style, behavior and ecosystem dynamics;
(3) A follow up program to monitor the Hg content and health of individuals that presents high levels of Hg on biological matrices.

## 5. Conclusions

The Hg exposure in the Amazonian population is a fact. The high level of Hg on hair revealed by this study shows that this population has been long-term exposed to this metal.

The data reveals that the studies focused on population that lives along the margins of the rivers and, of utmost importance, populations under risk of Hg exposure from gold mining activities, especially from Tapajós Basin located in the State of Pará.

## Figures and Tables

**Table 1:** Search strategies in electronic database.

Strategy	Keywords
#1	Mercury and hair and Amazon
#2	Methylmercury and hair and Amazon
#3	Mercury and Brazil and Amazonia
#4	Mercury and Amazon

**Table 2:** Characteristics of the included studies (*n =* 36).

Year	Author	Locality	State	*n*	Type of Population According to the Author	Population Specificity	Type of the Population According to the Present Study	Range Hg (µg g^−1^)	Mean Hg (µg g^−1^)
1998	Barbosa A.C. [16]	Fresco River	PA	28	Kayapo women	childbearing women	I	0.8–13.7	8.11
		Madeira River	RO	98	non-indigenous women	childbearing women	W	2.6–94.7	14.08
		Fresco River	PA	54	Kayapo children	nd	I	2.0–20.4	7.30
		Madeira River	RO	71	non-indigenous children	nd	C	0.8–44.4	10.82
1998	Kehrig H.A. [17]	Balbina Village	AM	53	total of population studied	nd	G	nd	6.54
				16	children	female	C	1.3–22.0	7.7
				12	children	male	C	2.5–11.4	5.3
				12	adults	female	A	2.2–15.5	7.4
				13	adults	male	A	1.2–12.2	5.5
1998	Barbosa A.C. [18]	Madeira River	RO	37	women	nd	W	2.0–37.2	14.3
				37	children	0.5–15 months	C	1.4–34.2	9.8
1999	Silva-Forsberg M.C. [19]	total (all the populations studied)	AM	154	total of population studied	0.2–66 y.o	G	5.76–171.24	75.46
		Acariquara, Rio Urubaxi		15	nd	0.3–56 y.o	G	14.37–146.25	69.18
		Tupuruquara, Rio Marie		57	nd	0.2–66 y.o	G	10.44–171.24	97.44
		Macuna, Rio Uneiuxi		17	nd	0.7–52 y.o	G	22.17–129.19	76.75
		Perseverança, Rio Negro		23	nd	0.2–65 y.o	G	15.77–122.32	65.72
		Ilha do Pinto. Rio Negro		12	nd	1.6–37 y.o	G	19.02–100.95	69.58
		Tapera. Rio Padauari		11	nd	2–59 y.o	G	19.20–55.59	37.48
		Tapereira. Rio Negro		10	nd	2–47 y.o	G	24.94–110.51	69.10
		Aldeia Maia. Rio Maia		7	nd	13–40 y.o	G	5.76–63.02	28.02
		Sitio Velho. Rio Marauia		2	nd	42–62 y.o	A	13.93–62.57	38.25
1999	Guimaraes J.R.D. [20]	Pracuuba Lake	AP	15	fishermen and their family	nd	nd	nd	16.7
		Duas Bocas Lake		15	fishermen and their family	nd	nd	nd	28
2000	Hacon S. [21]	Alta Floresta	MT	75	pregnant women	14–45 y.o	W	0.051–8.2	1.12
2000	Santos E.C.O. [22]	Brasília Legal	PA	220	total of population studied	0–>65 y.o	G	0.53–49.99	11.75
				30	nd	0–5 y.o	C	1.09–20.46	5.84
				68	nd	6–10 y.o	C	0.70–35.80	13.06
				33	nd	11–15 y.o	C	1.22–47.00	14.2
				12	nd	16–20 y.o	A	5.56–19.90	13.39
				10	nd	21–25 y.o	A	1.40–29.50	15.25
				9	nd	26–30 y.o	A	3.70–21.40	11.06
				16	nd	31–35 y.o	A	2.84–37.20	12.57
				12	nd	36–40 y.o	A	5.0–33.0	14.21
				1	nd	41–45 y.o	A	nd	11.7
				7	nd	46–50 y.o	A	1.02–14.24	7.06
				8	nd	51–55 y.o	A	3.57–49.99	11.53
				5	nd	56–60 y.o	A	0.53–7.07	4.93
				6	nd	61–65 y.o	A	5.01–15.94	11.33
				3	nd	>65 y.o	A	2.78–16.46	7.45
		São Luiz do Tapajós		327	total of population studied	0–>65 y.o	G	0.10–94.50	19.91
				75	nd	0–5 y.o	C	0.10–94.50	21.06
				74	nd	6–10 y.o	C	2.40–52.50	22.1
				51	nd	11–15 y.o	C	3.90–61.80	23.24
				21	nd	16–20 y.o	A	2.10–33.60	19.11
				21	nd	21–25 y.o	A	1.73–32.0	15.68
				15	nd	26–30 y.o	A	3.90–34.90	15.34
				15	nd	31–35 y.o	A	5.10–38.0	18.98
				15	nd	36–40 y.o	A	2.60–27.8	14.31
				14	nd	41–45 y.o	A	3.20–33.60	15.13
				8	nd	46–50 y.o	A	4.0–47.0	21.71
				4	nd	51–55 y.o	A	5.90–20.60	15.4
				6	nd	56–60 y.o	A	7.90–27.60	17.13
				3	nd	61–65 y.o	A	3.8–27.80	12.6
				5	nd	>65 y.o	A	3.20–20.80	12.6
		Santana de Ituqui		321	total of population studied	0–>65 y.o	G	0.40–11.60	4.33
				37	nd	0–5 y.o	C	0.50–8.50	3.67
				81	nd	6–10 y.o	C	0.40–10.9	4.44
				62	nd	11–15 y.o	C	2.0–11.6	4.47
				25	nd	16–20 y.o	A	2.5–9.60	5
				17	nd	21–25 y.o	A	1.30–7.10	3.34
				16	nd	26–30 y.o	A	1.70–9.20	4.69
				19	nd	31–35 y.o	A	1.90–9.60	5.36
				18	nd	36–40 y.o	A	1.20–6.0	3.44
				10	nd	41–45 y.o	A	2.70–6.80	4.18
				10	nd	46–50 y.o	A	1.90–6.40	4.02
				9	nd	51–55 y.o	A	2.30–9.0	5.39
				6	nd	56–60 y.o	A	3.10–6.90	4.37
				4	nd	61–65 y.o	A	1.90–9.0	4.15
				7	nd	>65 y.o	A	0.70–5.70	3.61
2000	Dolbec J. [23]	Cametá	PA	68	total of population studied	12–79 y.o	G	nd	10.8
2001	Barbosa A.C. [24]	Negro River	AM	73	children	<15 y.o	C	0.51–45.89	18.52
				76	adults	>15 y.o	A	1.66–59.01	21.4
2001	Harada M. [25]	Barreiras	PA	76	fisherman and family	1–67, mean 28 y.o	G	1.8–53.8	16.4
		Rainhas		12	fisherman and family	7–53, mean 31 y.o	G	3.1–34.5	14.1
		São Luiz do Tapajós		44	fisherman and family	3–47, mean 21 y.o	G	5.1–42.2	20.8
		Special group from Barreiras, Rainha and São Luiz do Tapajós		50	eligible subjects examined clinically that presented high level of Hg (>20 ppm) from March 1994 to February 1998	3–65, mean 25 y.o	G	5.1–42.7	23.6
2002	Crompton P. [26]	Jacareacanga	PA	205	total of population studied	general population, except children under 2 y.o	G	0.3–83.2	8.6
				nd	men	nd	M	nd	11.0
				nd	women	nd	W	nd	6.7
2003	Passos C.J. [27]	Brasília Legal	PA	26	adults women	23–62, mean 41 y.o	W	4.0–20.0	10.0
2003	Santos E.C.O. [28]	Guajará Mirim e Nova Mamoré	RO	910	total of studied population (Pakaanova indigenous)	0–>45 y.o	I	0.52–83.89	8.37
				57	nd	0–2 y.o	I	1.48–83.89	10.54
				115	nd	3–5 y.o	I	1.67–47.22	9.34
				152	nd	6–10 y.o	I	0.52–63.81	8.16
				114	nd	11–15 y.o	I	0.65–31.11	6.86
				177	nd	16–25 y.o	I	0.65–39.42	8.45
				114	nd	26–35 y.o	I	1.37–28.64	8.56
				50	nd	36–45 y.o	I	1.49–21.25	8.39
				131	nd	>45 y.o	I	1.37–25.84	7.84
2005	Dorea J.G. [29]	Teles Pires (Tapajós Basin)	PA	47	Kayabi indigenous	Kayabi community from Teles Pires	I	nd	12.8
				249	Munduruku indigenous	Munduruku community from Teles Pires	I	nd	3.4
2005	Santos E.C.O. [30]	São Gabriel da Cachoeira	AM	157	total of population studied	0–>40 y.o	G	0.30–83.11	13.02
				9	nd	0–5 y.o	C	1.01–14.40	5.71
				8	nd	6–10 y.o	C	2.05–15.00	7.35
				37	nd	11–20 y.o	NC	0.94–22.81	7.346
				45	nd	21–30 y.o	A	0.30–59.16	11.67
				33	nd	31–40 y.o	A	1.03–60.00	16.56
				26	nd	>40 y.o	A	2.41–83.11	22.88
		Barcelos		242	total of population studied	0–>40 y.o	G	0.07–52.04	9.671
				17	nd	0–5 y.o	C	0.83–25.89	7.46
				25	nd	6–10 y.o	C	0.76–27.46	6.85
				44	nd	11–20 y.o	NC	0.07–21.53	7.00
				62	nd	21–30 y.o	A	0.25–42.64	9.05
				27	nd	31–40 y.o	A	0.23–52.04	12.02
				67	nd	>40 y.o	A	2.60–32.86	12.67
2005	Tavares L.M.B. [31]	Riverine communities of Bocas de Conchas, Cuiabá Mirim, Estirão Cumprido and Porto Brandão located near of Barão de Melgaço	MT	72	riverine children	3–7 y.o	C	0.58–17.14	5.37
		Barão de Melgaço		114	urban children	3–7 y.o	C	0.38–7.57	2.08
2005	Klautau-Guimaraes M.N. [32]	Teles Pires	PA	65	total of population studied (Kayabi indigenous)	0–>61, mean 24.53 y.o	I	nd	14.75
				33	Kayabi indigenous	0–20 y.o	I	nd	17.86
				25	Kayabi indigenous	21–40 y.o	I	nd	11.97
				5	Kayabi indigenous	41–60 y.o	I	nd	14.35
				2	Kayabi indigenous	>61 y.o	I	nd	15.17
				117	total of population studied (Munduruku indigenous)	0–>61, mean 30.90 y.o	I	nd	nd
				34	Munduruku indigenous	0–20 y.o	I	nd	4.26
				56	Munduruku indigenous	21–40 y.o	I	nd	3.65
				20	Munduruku indigenous	41–60 y.o	I	nd	3.75
				7	Munduruku indigenous	>61 y.o	I	nd	3.72
2006	Alves M.F.A. [33]	total (all the riverine populations studied)	AM	105	total of adult studied population	18–50, mean 32 y.o	A	nd	35.4
		Mariuá (Negro River)		3	adults	nd	A	nd	24.9
		Marará (Negro River)		17	adults	nd	A	nd	27.3
		Piloto (Negro River)		25	adults	nd	A	nd	33.2
		Ponta da Terra (Cuiuni River)		12	adults	nd	A	nd	38.2
		São Luiz (Negro River)		21	adults	nd	A	nd	41.4
		Cumaru (Negro River)		16	adults	nd	A	nd	43.7
		Baturité (Negro River)		11	adults	nd	A	nd	33
		Manaus		105	total of studied population	18–50, mean 28 y.o	A	nd	1.0
2006	Bastos W.R. [34]	total (all the populations studied)	RO	713	total of population studied	nd	nd	5.99–150	15.22
		Calama		34	nd	nd	nd	0.50–22.48	9.02
		Boa Vitoria		3	nd	nd	nd	10.86–17.05	13.82
		Cujubim		12	nd	nd	nd	1.55–14.67	6.30
		Firmesa		4	nd	nd	nd	9.40–14.80	11.21
		Itacoa		6	nd	nd	nd	5.28–16.00	11.97
		Nazaré		64	nd	nd	nd	0.63–22.60	10.65
		Papagaios		13	nd	nd	nd	4.76–27.22	13.72
		Santa Rosa		19	nd	nd	nd	7.68–20.78	13.99
		São Carlos		15	nd	nd	nd	1.84–22.83	9.51
		Terra Caida		7	nd	nd	nd	5.01–14.61	9.61
		Sto Antônio do Pau Queimado		14	nd	nd	nd	5.87–26.86	14.69
		Puruzinho	AM	28	nd	nd	nd	4.57–28.27	14.83
		Livramento		15	nd	nd	nd	18.96–63.54	36.89
		Valparaiso		21	nd	nd	nd	2.98–82.38	18.93
		Auxiliadora		34	nd	nd	nd	1.12–22.78	9.34
		Curralinho		5	nd	nd	nd	10.70–34.49	19.69
		Nazaré do Retiro		15	nd	nd	nd	9.69–24.77	17.90
		Novos Prazeres		20	nd	nd	nd	2.77–24.28	11.90
		São Pedro		14	nd	nd	nd	6.61–28.00	15.77
		Barreiras do Manicoré		9	nd	nd	nd	1.45–23.04	10.82
		Cachoeirinha		14	nd	nd	nd	1.54–37.22	14.74
		São Lazaro		6	nd	nd	nd	2.50–23.37	9.48
		Maraca II		6	nd	nd	nd	8.57–15.69	11.37
		Vista Nova		4	nd	nd	nd	21.40–28.54	25.69
		Vista Alegre		17	nd	nd	nd	7.28–26.28	16.02
		Bom Suspiro		12	nd	nd	nd	6.43–30.06	16.29
		Carara		39	nd	nd	nd	4.18–34.71	18.13
		Miriti		16	nd	nd	nd	6.70–50.37	22.34
		São Sebastiao (Lago Lucio)		17	nd	nd	nd	6.61–18.52	12.84
		Boca do Carapanatuba		18	nd	nd	nd	3.43–19.22	10.45
		São Sebastiao do Tapuru		18	nd	nd	nd	20.43–150.00	62.76
		Moanenses		13	nd	nd	nd	3.26–20.49	12.73
		Três Casas		9	nd	nd	nd	5.62–70.70	33.07
		Boa Ventura		7	nd	nd	nd	4.73–35.79	16.55
		Auara Grande		19	nd	nd	nd	6.21–24.98	15.97
		Fazenda Tabocal		2	nd	nd	nd	0.50–1.50	1.00
		Remanso		12	nd	nd	nd	8.36–29.02	18.16
		Arapapa		7	nd	nd	nd	10.43–21.33	16.56
		Axinim		13	nd	nd	nd	3.27–23.02	8.65
		Espirito Santo		18	nd	nd	nd	3.51–21.28	12.47
		Santa Maria		7	nd	nd	nd	6.70–16.84	9.28
		Caicara		23	nd	nd	nd	1.94–17.98	10.04
		Paquique		6	nd	nd	nd	7.49–11.57	9.23
		Uricurituba		46	nd	nd	nd	0.36–19.12	9.09
		Santa Rosa II		12	nd	nd	nd	5.81–16.89	11.65
2006	Fillion M. [35]	Tapajós (São Luiz do Tapajós, Nova Canaã, Santo Antônio, Mussum, Vista Alegre, Açaituba)	PA	251	adults	15–89, mean 35.2 y.o	A	0.21–77.2	17.8
2007	Passos C.J. [36]	Tapajós (São Luiz do Tapajós, Nova Canaã, Santo Antônio, Ipaupixuna, Novo Paraiso, Teca, Timbó, Açaituba, Campo Alegre, Sumauma, Vista Alegre, Mussum, Santa Cruz)	PA	449	adults	15–89, mean 38.6 y.o	A	0.2–58.3	16.8
2007	Pinheiro M.C.N. [37]	Panacauera	PA	8	children	0–1 y.o	C	0.39–4.66	1.11
				13	children	2–6 y.o	C	0.65–5.16	2.27
				15	children	7–12 y.o	C	0.86–9.46	2.99
		Barreiras (Tapajós Basin)		17	children	0–1 y.o	C	1.80–15.70	5.35
				45	children	2–6 y.o	C	1.43–23.60	6.21
				22	children	7–12 y.o	C	1.63–14.50	6.72
		São Luiz do Tapajós		11	children	0–1 y.o	C	1.99–30.30	5.97
				23	children	2–6 y.o	C	2.76–53.80	13.22
				14	children	7–12 y.o	C	1.34–38.80	10.83
2008	Passos C.J.S. [38]	Tapajós (São Luiz do Tapajós, Nova Canaã, Santo Antônio, Vista Alegre, Mussum, Açaituba)	PA	256	adults	15–89, mean 35.3 y.o	A	0.2–58.3	17.9
2009	Fillion M. [39]	Tapajós (São Luiz do Tapajós, Nova Canaã, Santo Antônio, Mussum, Vista Alegre, Açaituba, Santa Cruz, Sumauma, Campo Alegre, Ipaupixuna, Novo Paraiso, Curi-Teca, Curi-Timbó)	PA	456	adults	15–>65 y.o	A	0.2–77.7	17.8
2010	Grotto D. [40]	Tapajós	PA	108	total of population studied	mean 41.1 y.o	nd	1–57.8	13.7
				54	men	nd	M	nd	11.5
				54	women	nd	W	nd	8.8
2010	Bortoli M.C. [41]	Novo Airão	AM	55	women	mean 32.3 y.o	W	0.04–18.67	5.67
2012	Barcelos G.R.M. [42]	Tapajós	PA	144	adults	15–83, mean 43 y.o	A	1–43.3	10.4
2012	Dutra M.D.S. [43]	Itaituba	PA	90	children	population from urban area, samples collected in 2004	C	nd	1.01
				47	children	population from urban area, samples collected in 2006	C	nd	1.18
				90	children	population from urban area, samples collected in 2010	C	nd	1.18
2012	Farias L.A. [44]	Manaus	AM	201	children	2–7 y.o	C	0.02–34.4	1.93
2013	Khoury E.D.T. [45]	Barreiras	PA	78	general population	13–53 y.o	G	nd	8.66
		São Luiz do Tapajós		30	general population	13–53 y.o	G	nd	9.19
		Furo do Maracujá		49	general population	13–53 y.o	G	nd	0.73
2014	Rocha A.V. [46]	Demarcação—Machado River	RO	10	children	3–9 y.o	C	nd	3.57
		Gleba do Rio Preto		10	children	3–9 y.o	C	nd	6.24
2015	Faial K. [47]	Itaituba	PA	6	male	0–2 y.o	C	4.14–9.79	6.85
				6	male	3–5 y.o	C	16.01–23.80	19.57
				10	male	6–10 y.o	C	12.59–24.93	18.58
				6	male	11–15 y.o	C	5.83–15.57	13.08
				7	male	16–20 y.o	A	17.82–24.16	20.87
				4	male	21–25 y.o	A	9.42–20.09	15.52
				nd	male	26–30 y.o	A	nd	nd
				3	male	31–35 y.o	A	8.55–10.83	9.69
				5	male	36–40 y.o	A	10.92–20.03	15.29
				1	male	41–45 y.o	A	14.81–14.81	14.81
				1	male	46–50 y.o	A	2.07–2.07	2.07
				1	male	51–55 y.o	A	13.89–13.89	13.89
				3	male	56–60 y.o	A	14.00–14.16	14.08
				5	male	>60 y.o	A	12.04–21.47	14.57
				4	female	0–2 y.o	C	8.25–12.89	10.38
				7	female	3–5 y.o	C	12.20–19.29	15.83
				7	female	6–10 y.o	C	11.46–23.21	15.84
				8	female	11–15 y.o	C	8.41–21.71	13.12
				5	female	16–20 y.o	A	13.52–22.01	16.74
				14	female	21–25 y.o	A	6.33–17.66	11.22
				7	female	26–30 y.o	A	4.84–10.6	7.16
				8	female	31–35 y.o	A	4.95–20.78	13.52
				3	female	36–40 y.o	A	7.30–15.73	11.09
				3	female	41–45 y.o	A	14.69–23.02	18.62
				3	female	46–50 y.o	A	7.31–14.98	10.27
				4	female	51–55 y.o	A	15.52–18.52	17.02
				1	female	56–60 y.o	A	14.29–14.29	14.29
				9	female	>60 y.o	A	14.60–27.02	20.39
2015	Castilhos Z. [48]	São Chico	PA	172	nd	nd	nd	0.14–35.90	3.44
		Creporizinho		146	nd	nd	nd	0.23–10.49	2.25
2015	Hoshino A. [49]	Lago do Puruzinho	AM	58	general population	1–47, mean 17.3 y.o	G	nd	12.78
2016	Rocha A.V. [50]	Porto Velho	RO	200	women	18–48, mean 26.60 y.o	W	nd	0.60
2016	Carvalho L.V.B. [51]	Belmont	RO	42	nd	mean 11.3 y.o	nd	nd	2.71
		Cunia		52	nd	mean 11.3 y.o	nd	nd	7.18
				11.827					

Legend. NC: not included for map classification, nd: not determined; G: general population; C: children; A: adults; W: women; M: men; I: Indigenous, >older than. y.o: year(s) old.
